# Single Hormone Receptor-Positive Metaplastic Breast Cancer: Similar Outcome as Triple-Negative Subtype

**DOI:** 10.3389/fendo.2021.628939

**Published:** 2021-04-23

**Authors:** Jinqian Mao, Jin Hu, Yanting Zhang, Jian Shen, Fang Dong, Ximeng Zhang, Jie Ming, Tao Huang, Xiaoqin Run

**Affiliations:** ^1^Department of Vascular Surgery, Union Hospital, Tongji Medical College, Huazhong University of Science and Technology, Wuhan, China; ^2^Department of Breast and Thyroid Surgery, Union Hospital, Tongji Medical College, Huazhong University of Science and Technology, Wuhan, China; ^3^Department of Ultrasound, Union Hospital, Tongji Medical College, Huazhong University of Science and Technology, Wuhan, China; ^4^Department of Pancreatic Surgery, Union Hospital, Tongji Medical College, Huazhong University of Science and Technology, Wuhan, China

**Keywords:** metaplastic breast cancer, hormonal receptor, HER2, prognosis, SEER database

## Abstract

**Background:**

Metaplastic breast cancer (MBC) is a rare and aggressive subtype of the breast. To understand the characteristics and prognosis of single hormone receptor-positive (HR+) MBC (estrogen receptor-positive [ER+]/progesterone receptor-negative [PR-] and ER-/PR+), we compared these tumors to double HR+ tumors as well as HR- tumors.

**Patients and Methods:**

The Surveillance, Epidemiology, and End Results database was used to analyze MBC between 1975 and 2016. The effect of HR status was evaluated using a multivariate Cox regression model.

**Results:**

We included 3369 patients with a median follow-up time of 42 months (range 0-322 months). In this study, 280 (8.3%) cases were double HR+ tumors, 2597 (77.1%) were double HR- tumors, and 492 (14.6%) cases were single HR+ tumors, of which 159 (4.7%) cases were ER-/PR+ tumors and 333 (9.9%) were ER+/PR- tumors. On multivariate Cox analysis, the prognosis was related to age, race/ethnicity, tumor grade, TNM stage, and surgery. HR status remained no impact on breast cancer-specific survival (BCSS). In the Kaplan-Meier curve, HR status was not associated with better BCSS or overall survival (OS). In patients without HER2 overexpression, the BCSS and OS of ER+/PR- and ER-/PR+ tumors were not significantly different from that of ER-/PR- and ER+/PR+ tumors. The difference remains no significant in patients with HER2 overexpression.

**Conclusions:**

In comparison with both ER-/PR- and ER+/PR+ tumors, we have identified clinically and biologically distinct features of single HR+ tumors. In patients with or without HER2 overexpression, the prognosis of single HR+ tumors was similar to ER-/PR- and ER+/PR+ tumors.

## Introduction

Metaplastic breast cancer (MBC) is a rare and aggressive subtype accounting for <1% of all breast cancers (1). Previous studies have reported histologic MBC characterized by either homogenous or mixed components (2–6). MBC was not identified as a unique pathological type by the World Health Organization until 2000 (7). Since then, as pathologists’ understanding of MBC has considerably improved, the incidence has also increased (8). However, given its rarity, the clinical characteristics and prognosis of single hormonal receptor-positive MBC (single HR+ MBC, ER+/PR-, and ER-/PR+) are unclear.

In the National Comprehensive Cancer Network (NCCN) breast cancer guidelines, the management of MBC is similar to that of invasive ductal carcinoma (IDC) (9). However, MBC is characterized by larger tumor size, lesser regional node involvement, and higher tumor grade than breast cancers with more common histology (10–12). The pathway of metaplastic cancer metastasis was hematogenous but not lymphatic spread (13). A previous study with data from 2001 to 2010 of the Surveillance, Epidemiology, and End Results (SEER) database found that patients with stage I–III MBC had significantly worse 5-year breast cancer-specific survival (BCSS) than those with synchronous IDC (14). Some studies reported that MBC is chemorefractory, regardless of whether the included patients received neoadjuvant or adjuvant settings (8, 15–17). Although the common molecular subtype is the triple-negative (TN) phenotype in MBC, HR+ and human epidermal growth receptor 2 positive (HER2+) tumors do exist (18). A population-based study reported that HR status was not associated with survival of metaplastic carcinoma, which was different from IDC and infiltrating lobular carcinomas (19).

Although the technique of immunohistochemistry has now considerably improved, the incidence of MBC with estrogen receptor-negative (ER-)/progesterone receptor-positive (PR+) phenotype has not decreased (20). Generally, HR+ breast cancers have a favorable prognosis. To understand the characteristics and prognosis of single HR+ MBC, we compared these tumors to double HR+ tumors (ER+/PR+) as well as HR- tumors (ER-/PR-) by using the database of the whole population.

## Materials and Methods

### Patients

Data were retrieved from the SEER database and included all cases of pathologically confirmed MBC diagnosed between 1975 and 2016. This database collects data on cancer incidence, demographics and clinicopathologic data, management, and survival from 18 population-based cancer registries. According to the third edition of the International Classification of Diseases for Oncology (ICD-0-3), carcinoma histology was identified in metaplastic cancers with ICD-0-3 codes: 8560, 8562, 8570–8572, 8575, and 8980–8982 (19). The inclusion criteria were as follows: female sex; age ≥18 years; breast cancer as first and the only cancer diagnosis; unilateral breast cancer; histologically or cytologically confirmed diagnosis (instead of autopsy-confirmed); available information regarding survival time and HR status; and stage exception of T0 and Tis. Accordingly, 3369 patients were finally enrolled.

### Demographics and Clinicopathologic Features

The demographic parameters included age at diagnosis; race/ethnicity recorded in the SEER database (White, Black, other); and insurance status. The clinicopathologic parameters included tumor grade; tumor size (T1, T2, T3, T4); regional node status (N0, N1, N2, N3); chemotherapy (CT); radiotherapy (RT); type of surgery (no surgery, lumpectomy, mastectomy); and biomarker profile (ER, PR, HER2). The definition of TNM (T-tumor, N-node, and M-metastasis) stage was according to the sixth/seventh edition of the Union for International Cancer Control/American Joint Committee on Cancer Pathologic Staging System. According to the SEER, HR status was stratified as single HR+, double HR+ tumors, and double HR- tumors.

The primary clinical outcome was BCSS, defined as the date of diagnosis to the date of death from breast cancer. The secondary clinical outcome was overall survival (OS), defined as the date of diagnosis to the date of death from any cause.

### Detection of ER, PR, and HER2

In the SEER database, in cases where ER/PR is reported on more than one tumor specimen, the highest value is recorded. If any sample is positive, that record as positive. If neoadjacent therapy was received, the assay was recorded from tumor specimens prior to neoadjuvant therapy. If neoadjuvant therapy was given and there were no ER/PR results from pre-treatment specimens, these findings were reported from post-treatment specimens. If ER/PR was positive on an *in situ* specimen and ER/PR was negative on all tested invasive specimens, code ER/PR was considered negative. If ≥1% cells stained positive, the test results were considered positive. HER2 positivity was defined as an intensity of 3+ by IHC, while a score of 2+ was interpreted as equivocal. A negative test was defined as staining with a score of 0/1+. For equivocal stating, silver *in situ* hybridization (SISH) or fluorescence *in situ* hybridization (FISH) were performed; the results were positive for HER2 amplification when the ratio of HER2 to CEP17 was >2.2. We provided four MBC patients with different ER/PR phenotype (**Figure S1**).

### Statistical Analysis

The χ2 test was carried out to analyze the differences between groups. The Cox proportional hazards model was used to assess the risk factors related to BCSS. Survival curves were constructed using the Kaplan–Meier method. Hazard ratios were presented with 95% confidence intervals (CIs). All statistical analyses were performed using SPSS statistical software (version 24.0; IBM Corporation, Armonk, NY, USA), and P <0.05 was considered to indicate statistical significance.

## Results

### Patient Characteristics

Of the 4672 MBC patients in the SEER registry, our final sample comprised 3369 patients. In this study, 280 (8.3%) patients had double HR+ tumors, 2597 (77.1%) had double HR- tumors, and 492 (14.6%) had single HR+ tumors, of which 159 (4.7%) cases were ER-/PR+ tumors and 333 (9.9%) were ER+/PR- tumors. The median age of the entire cohort was 61 years (range, 20–89 years). Most patients were white women (n=2565, 76.1%) and had poor differentiation (n=2274, 67.5%). In patients with available tumor size information, 46.0% were stage T2. A total of 3199 (95.0%) and 170 (5.0%) patients had stage I–III and stage IV disease, respectively. In addition, 2450 (72.7%), 576 (17.1%), 131 (3.9%), and 75 (2.2%) patients had N0, N1, N2, and N3 stage disease, respectively. A total of 1194 deaths were recorded, including 791 breast cancer related-deaths.

The clinicopathological characteristics of the four subtypes are summarized in **Table 1**. Compared with ER-/PR- tumors, ER+/PR- tumors were not significantly different with respect to ethnicity, tumor grade, tumor stage, and CT, but ER+/PR- tumors exhibited more regional node involvement (P = 0.004). However, compared with ER+/PR+ tumors, the clinicopathological characteristics of ER+/PR- tumors did not show a significant difference. ER-/PR+ tumors were found more in Black women (ER-/PR+ 25.2% vs. ER-/PR- 17.4%, P = 0.021) and had higher tumor grade (P = 0.010) than ER-/PR- tumors. Further, ER-/PR+ tumors were also found in more black women (ER-/PR+ 25.2% vs. ER-/PR- 13.4%, P = 0.012), had higher tumor grade (P = 0.003), and received more CT treatment (ER-/PR+ 67.3% vs. ER-/PR- 56.8%, P = 0.030) than ER+/PR+ tumors. There was no difference in stage (P = 0.139) or type of surgery (P = 0.288). Furthermore, there was no difference in the expression of HER2 (P = 0.831). Both ER-/PR+ and ER+/PR- tumors had similar HER2 overexpression to ER+/PR+ tumors (P = 0.831). However, ER-/ER+ tumors showed higher HER2 overexpression than ER-/PR- tumors (P = 0.028). The characteristics of single HR+ tumors were more distinct in HER2-negative tumors than in HER2 overexpressing tumors. (**Tables S1** and **S2**)

**Table 1:** Characteristics in MBC patients with ER-/PR-, ER+/PR-, ER-/PR+, and ER+/PR+ tumors.

Variables	ER-/PR-	ER+/PR-	ER-/PR+	ER+/PR+
Age (years)	61.25 ± 13.95	61.63 ± 14.07	60.21 ± 15.71	60.40 ± 15.45
Follow-up time (median, months)	43 (0-322)	34 (0-321)	45 (0-320)	42 (0-273)
Race (n, %)				
Black	421 (16.2)	55 (16.5)	36 (25.2)	34 (12.1)
White	1998 (76.9)	240 (72.1)	107 (74.8)	220 (78.6)
Other	178 (6.9)	38 (11.4)	16	26 (9.3)
Insurance (n, %)				
No	928 (35.7)	55 (16.5)	44 (27.7)	97 (34.6)
Yes	1669 (64.3)	278 (83.5)	115 (72.3)	183 (65.4)
Grade (n, %)				
Undifferentiated	141 (5.4)	13 (3.9)	6 (3.8)	9 (3.2)
Poorly differentiated	1758 (67.7)	228 (68.5)	120 (75.4)	168 (60.0)
Moderately differentiated	261 (10.1)	43 (12.9)	17 (10.7)	50 (17.9)
Well differentiated	91 (3.5)	11 (3.3)	9 (5.7)	14 (5.0)
Unknown	346 (13.3)	38 (11.4)	7 (4.4)	39 (13.9)
Tumor size (n, %)				
T1	642 (24.7)	83 (24.9)	47 (29.6)	82 (29.3)
T2	1205 (46.4)	152 (45.7)	75 (47.2)	118 (42.1)
T3	411 (15.8)	41 (12.3)	21 (13.2)	37 (13.2)
T4	211 (8.2)	41 (12.3)	13 (8.2)	27 (9.6)
Unknown	128 (4.9)	16 (4.8)	3 (1.8)	16 (5.7)
Regional node status (n, %)				
N0	1927 (74.2)	217 (65.2)	120 (75.5)	186 (66.4)
N1	431 (16.6)	71 (21.3)	26 (16.4)	48 (17.2)
N2	89 (3.4)	18 (5.4)	3 (1.9)	21 (7.5)
N3	47 (1.8)	13 (3.9)	4 (2.5)	11 (3.9)
Unknown	103 (4.0)	14 (4.2)	6 (3.7)	14 (5.0)
TNM stage (n, %)				
I-III	2356 (90.7)	294 (88.3)	146 (91.8)	257 (91.8)
IV	129 (5.0)	24 (7.2)	9 (5.7)	8 (2.9)
Unknown	112 (4.3)	15 (4.5)	4 (2.5)	15 (5.3))
HER2				
Positive	1154 (44.4)	211 (63.4)	74 (46.5)	122 (43.6)
Negative	65 (2.5)	13 (3.9)	10 (6.3)	14 (5.0)
Unknown	1378 (53.1)	109 (32.7)	75 (47.2)	144 (51.4)
Chemotherapy (n, %)				
No	937 (36.1)	119 (35.7)	52 (32.7)	121 (43.2)
Yes	1660 (63.9)	214 (64.3)	107 (67.3)	159 (56.8)
Radiotherapy (n, %)				
No	1474 (56.8)	188 (56.5)	79 (49.7)	153 (54.6)
Yes	1123 (43.2)	145 (43.5)	80 (50.3)	127 (45.4)
Type of surgery (n, %)				
No	201 (7.7)	29 (8.7)	9 (5.7)	24 (8.6)
Lumpectomy	1045 (40.2)	133 (39.9)	73 (45.9)	110 (39.3)
Mastectomy	1351 (52.0)	171 (51.4)	77 (48.4)	146 (52.1)

MBC, metaplastic breast cancer; ER, estrogen receptor; PR, progesterone receptor.

### Prognostic Factors for MBC

We further analyzed the independent prognostic factors associated with BCSS using the multivariate Cox proportional hazards model. HR status was not an independent prognostic factor related to better BCSS (hazard ratio: 0.839; 95%CI: 0.679–1.036; P = 0.102). Patients with stage IV disease had a worse prognosis than patients with stage I–III disease (hazard ratio: 7.594; 95%CI: 6.308–9.289; P < 0.001). In addition, patients could not benefit from CT (hazard ratio: 0.993; 95%CI: 0.839–1.176; P = 0.937) and RT (hazard ratio: 0.895; 95%CI: 0.552–1.535; P = 0.687). Patients who underwent mastectomy had worse prognosis than those who underwent lumpectomy (hazard ratio: 2.131; 95%CI: 1.795–2.530; P < 0.001). Furthermore, age, race/ethnicity, and tumor grade were independent indicators for BCSS (**Table 2**).

**Table 2:** Prognostic factors for BCSS in our study cohort.

Variables	Univariate analysis		P	Multivariate analysis		P
	HRs	95% CI		HRs	95% CI	
Age	1.010	1.005-1.015	<0.001	1.016	1.010-1.021	< 0.001
Race (n, %)						
Black	1	[Reference]		1	[Reference]	
White	0.754	0.631-0.901	0.002	0.788	0.657-0.944	0.010
Other	0.640	0.465-0.881	0.003	0.725	0.525-1.001	0.051
Insurance (n, %)						
No	1	[Reference]		1	[Reference]	
Yes	1.009	0.873-1.166	0.903	1.051	0.904-1.222	0.515
HR status						
ER-/PR-	1	[Reference]		1	[Reference]	
ER+/PR-	0.966	0.753-1.239	0.788	0.914	0.712-1.172	0.478
ER-/PR+	0.896	0.673-1.259	0.896	0.942	0.670-1.326	0.734
ER+/PR+	0.985	0.766-1.268	0.907	0.934	0.725-1.203	0.597
Grade (n, %)						
Undifferentiated	1	[Reference]		1	[Reference]	
Poorly differentiated	0.683	0.527-0.886	0.004	0.805	0.619-1.047	0.105
Moderately differentiated	0.358	0.247-0.520	<0.001	0.416	0.285-0.607	<0.001
Well differentiated	0.243	0.131-0.451	<0.001	0.333	0.179-0.619	0.001
Unknown	0.803	0.594-1.086	0.154	0.810	0.598-1.099	0.176
TNM stage (n, %)						
I-III	1	[Reference]		1	[Reference]	
IV	10.378	8.554-12.592	<0.001	7.594	6.308-9.289	<0.001
Chemotherapy (n, %)						
No	1	[Reference]		1	[Reference]	
Yes	1.035	0.895-1.196	0.642	0.993	0.839-1.176	0.937
Radiotherapy (n, %)						
No	1	[Reference]		1	[Reference]	
Yes	0.798	0.469-1.357	0.404	0.895	0.552-1.535	0.687
Type of Surgery (n, %)						
Lumpectomy	1	[Reference]		1	[Reference]	
Mastectomy	2.438	2.057-2.889	<0.001	2.131	1.795-2.530	<0.001
No	4.173	3.299-5.278	<0.001	3.092	2.418-3.952	<0.001

BCSS, breast cancer-specific survival; HR, hormonal receptor; HRs, hazard ratios; CI, confidence interval; ER, estrogen receptor; PR, progesterone receptor.

### Survival Analysis of Single Hormone Receptor-Positive MBC

**Figure 1:**
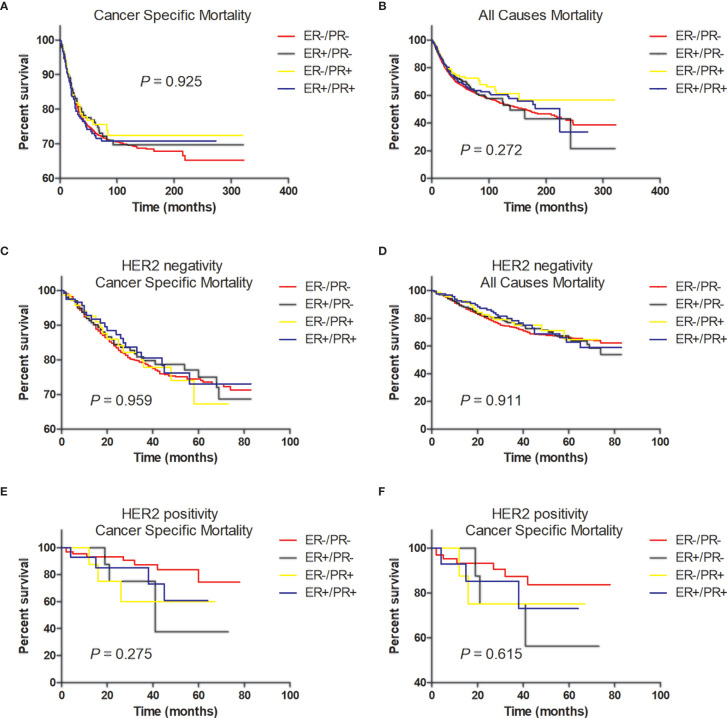
Tumor survival based on hormone receptor status. **(A)** Breast cancer-specific survival (BCSS) and **(B)** overall survival (OS) of all patients; **(C)** BCSS and **(D)** OS of patients with HER2-negative tumors; **(E)** BCSS and **(F)** OS of patients with HER2-positive tumors.

In multivariate analysis, in patients with or without HER2 overexpression, HR status was not associated with better BCSS or OS (**Tables 3**, **4**). Survival curves were plotted using the Kaplan–Meier curve. HR status was neither associated with BCSS nor OS (**Figures 1A, B**). In patients without HER2 overexpression, the BCSS and OS of ER+/PR- and ER-/PR+ tumors were not significantly different from those of ER-/PR- and ER+/PR+ tumors (**Figures 1C, D**). In patients with HER2 overexpression, the prognosis of ER+/PR- and ER-/PR+ tumors was not significantly different from those of ER-/PR- and ER+/PR+ tumors (**Figures 1E, F**).

**Table 3:** Multivariate analysis of BCSS and OS in 1561 women with HER2-negative MBC.

		B coefficients	Standard error	Wald	*P*	HRs	95% CI
BCSS	ER-/PR- vs. ER+/PR-	-0.131	0.179	0.534	0.465	0.877	0.618-1.246
	ER-/PR- vs. ER-/PR+	0.254	0.271	0.881	0.348	1.289	0.758-2.192
	ER-/PR- vs. ER+/PR+	-0.113	0.238	0.224	0.636	0.893	0.561-1.424
	ER+/PR+ vs. ER+/PR-	-0.018	0.281	0.004	0.949	0.982	0.566-1.705
	ER+/PR+ vs. ER-/PR+	0.367	0.348	1.112	0.292	1.443	0.730-2.852
OS	ER-/PR- vs. ER+/PR-	-0.058	0.153	0.143	0.706	0.944	0.700-1.273
	ER-/PR- vs. ER-/PR+	0.023	0.252	0.008	0.927	1.023	0.624-1.677
	ER-/PR- vs. ER+/PR+	-0.113	0.205	0.305	0.581	0.893	0.598-1.334
	ER+/PR+ vs. ER+/PR-	0.055	0.241	0.053	0.818	1.057	0.659-1.696
	ER+/PR+ vs. ER-/PR+	0.136	0.314	0.188	0.665	1.146	0.619-2.122

BCSS, breast cancer-specific survival; OS, overall survival; MBC, metaplastic breast cancer; HER2, human epidermal growth factor receptor 2; ER, estrogen receptor; PR, progesterone receptor; HRs, hazard ratios; CI, confidence interval. Adjusted for age, race, insurance, T stage, N stage, nuclear grade, and treatment.

**Table 4:** Multivariate analysis of BCSS and OS in 102 women with HER2-positive MBC.

		B coefficients	Standard error	Wald	*P*	HRs	95% CI
BCSS	ER-/PR- vs. ER+/PR-	-0.093	1.040	0.008	0.929	0.912	0.119-7.001
	ER-/PR- vs. ER-/PR+	-0.710	1.270	0.313	0.576	0.492	0.041-5.927
	ER-/PR- vs. ER+/PR+	1.074	1.087	0.976	0.323	2.927	0.348-24.657
	ER+/PR+ vs. ER+/PR-	-1.167	1.424	0.671	0.413	0.311	0.019-5.072
	ER+/PR+ vs. ER-/PR+	-1.784	1.543	1.337	0.248	0.168	0.008-3.457
OS	ER-/PR- vs. ER+/PR-	-0.093	1.040	0.008	0.929	0.912	0.119-7.001
	ER-/PR- vs. ER-/PR+	-0.710	1.270	0.313	0.576	0.492	0.041-5.927
	ER-/PR- vs. ER+/PR+	1.074	1.087	0.976	0.323	2.927	0.348-24.657
	ER+/PR+ vs. ER+/PR-	-1.167	1.424	0.671	0.413	0.311	0.019-5.072
	ER+/PR+ vs. ER-/PR+	-1.784	1.543	1.337	0.248	0.168	0.008-3.457

BCSS, breast cancer-specific survival; OS, overall survival; MBC, metaplastic breast cancer; HER2, human epidermal growth factor receptor 2; ER, estrogen receptor; PR, progesterone receptor; HRs, hazard ratios; CI, confidence interval.

Adjusted for age, race, insurance, T stage, N stage, nuclear grade, and treatment.

## Discussion

In the current study, we evaluated tumor response to treatment with CT, RT, and surgery and compared differences in the clinical process, tumor characteristics, and prognosis among the four subtypes, namely ER-/PR-, ER+/PR-, ER-/PR+, and ER+/PR+. We found that CT and RT could not improve the prognosis of MBC. Patients that underwent mastectomy had a worse prognosis than those that underwent lumpectomy. Of concern was the finding that HR status was not associated with a better prognosis in the entire cohort. In patients with or without HER2 overexpression, the prognosis of single HR+ tumors was similar to that of ER-/PR- and ER+/PR+ tumors.

The data presented in this paper represent the largest cohort of patients with MBC, and this is the first descriptive report on the survival prognosis of MBC related to single HR status. For traditional breast cancer, patients with ER+/PR+ tumors had a better prognosis than those with ER+/PR- tumors, who in turn had a better prognosis than patients with ER-/PR- tumors (21). However, to our knowledge, no previous research has investigated the prognosis of single HR+ tumors in case of metaplastic carcinoma.

Ahmed et al. (20) reported that ER-/PR+ breast cancers exist, but are very rare. Itoh et al. (22) reported that among the ER-/PR+ patients, 65% of them were basal-like tumors. Bae et al. (23) pointed out that in single HR+ breast cancers, the ER+/PR- subtype accounts for 10%–15% of all breast cancers, while the ER-/PR+ subtype accounts for 2–4% of all breast cancers. Based on the SEER records, the frequency of the ER-/PR+ phenotype in our series was 4.7%. On the one hand, the results of immunohistochemistry from the SEER database were confirmed by pathologists. On the other hand, MBC tended to have poor differentiation accounting for 67.5% of all cases. In addition, Weigelt et al. (24) showed that MBCs are basal-like breast cancers. These reports propose that ER-/PR+ breast cancers are a biologically and clinically distinct subtype.

Although TN-subtype is the most common in MBC, the HR+ subtype also occurs (18). A study by Wright et al. (19) including 2,338 MBC cases concluded that contrary to traditional breast cancers, HR+ MBC did not have superior clinical outcomes. In our study, 2597 (77.1%), 333 (9.9%), 159 (4.7%), and 280 (8.3%) patients expressed ER-/PR-, ER+/PR-, ER-/PR+, and ER+/PR+, respectively. There was no difference in the prognosis among the four subtypes. In addition, He et al. (25) concluded that patients with TN-subtype had a worse prognosis than those with non-TN MBC. However, our study results showed that regardless of HER2 overexpression, the prognosis of ER+/PR- and ER-/PR+ tumors were not significantly different from those of ER-/PR- and ER+/PR+ tumors. The results using multivariate analysis may be more convincing than those obtained with Kaplan–Meier analysis that they using.

Although the rate of adjuvant CT was quite high (63.9% in ER-/PR-, 64.3% in ER+/PR-, 67.3% in ER-/PR+, and 56.8% in ER+/PR+; P=0.081), there was no significant difference among the four subtypes in the entire cohort or in patients with or without HER2 overexpression. However, previous research has shown that the response rate of MBC to CT regimens was relatively low. MBC might be a type of basal breast cancer, characterized by higher grade and more rapid growth (24, 26–28). The expression levels of ER, PR, and HER-2 receptor in MBC cells were lower than that of IDC, while the expression levels of Ki-67 and p-53 were higher (29, 30). In MBC patients, DNA repair pathways such as TOP2A, PTEN, and BRCA1 showed downregulation upon genomic profiling. These findings might explain the low incidence of lymph node metastasis and resistance to conventional CT regimens. This may be one of the causes of the poor prognosis of patients with MBC.

A recent retrospective analysis showed that RT was related to improvements in OS and BCSS (25). However, some authors pointed out that the role of RT in the prognosis of MBC was related to the types of surgical methods. As we know, post-lumpectomy RT is a standard component of lumpectomy for treating IDC to minimize local recurrence. Dave et al. (31) and Yu et al. (32) found that RT was beneficial for MBC patients undergoing a lumpectomy, but not a total mastectomy. Additionally, a few studies illustrated that the role of RT in prognosis was related to clinical characteristics of MBC besides the types of surgical methods. However, our study found that receipt of RT was not an independent factor for improved survival.

Notably, mastectomy was performed more often for patients with MBC, likely due to the presentation of larger tumors than those with other types of breast cancer. Tseng and Martinez explained that mastectomy or lumpectomy had no effect on OS or disease-specific survival for patients with MBC (33). In our study, the rate of patients receiving mastectomy was higher than that of patients receiving lumpectomy (51.8% vs. 40.4%), but mastectomy was an independent risk factor for BCSS. This may be another cause for poor prognosis with MBC.

Although detailed endocrine treatment strategies were not available in this analysis, previous studies have reported that the prognosis of HR+ patients receiving antiestrogen therapy showed no difference in outcome as compared to that of patients who did not receive antiestrogen therapy (8, 16, 34). The prognosis of single HR+ MBC is as poor as that of TN-MBC, which may be due to some factors.

Our study has some limitations. First, the retrospective nature of the study may have resulted in some selection bias. Second, detailed chemotherapy regimens, radiotherapy information, and endocrine treatment strategies could not be available from the SEER database; hence, a further case-control analysis could not be performed. However, we believe that our results will help researchers to understand the role of single hormonal receptor status in the prognosis of MBC.

## Conclusion

We assessed a large cohort of patients with metaplastic breast cancer and found that HR status was not associated with prognosis. Furthermore, regardless of HER2 overexpression, the prognosis of ER+/PR- and ER-/PR+ tumors was not significantly different from those of ER-/PR- and ER+/PR+ tumors. When patients diagnosed with this rare and aggressive tumor were treated with surgery, physicians need to be careful with selecting the type of surgery. Furthermore, the role of anti-hormone therapy in HR+ MBC may need to be further investigated.

## Data Availability Statement

The original contributions presented in the study are publicly available. This data can be found here: https://seer.cancer.gov/data

## Ethics Statement

This study was exempt from the approval processes of the Institutional Review Boards because the SEER database patient information is de-identified. Also, a patient consent form was not applicable.

## Author Contributions

XR, TH, and JMi contributed to the conception and design of the study. JH and JMa organized the database. YZ, JS, FD, and XZ performed the statistical analysis. JH wrote the first draft of the manuscript. JMa, YZ, JS, FD, and XZ wrote sections of the manuscript. All authors contributed to the article and approved the submitted version.

## Funding

This work was supported by the National Natural Science Foundation of China (Grant No. 81672611).

## Conflict of Interest

The authors declare that the research was conducted in the absence of any commercial or financial relationships that could be construed as a potential conflict of interest.

## Supplementary Material

Supplementary Table 1 Clinicopathologic characteristics of patients with HER2-negative tumors.

Supplementary Table 2 Clinicopathologic characteristics of patients with HER2-positive tumors.

Supplementary Figure 1 Four MBC patients with different estrogen receptor (ER)/progesterone receptor (PR) phenotype. a, H&E, ×200; b, ER (-),×200; c, PR (-),×200; d, H&E, ×200; e, ER (+),×200; f, PR (+),×200; g, H&E, ×200; h, ER (-),×200; i, PR (+),×200; j, H&E, ×200; k, ER (+),×200; m, PR (-),×200.
