# Supplementary material for: Single Hormone Receptor-Positive Metaplastic Breast Cancer: Similar Outcome as Triple-Negative Subtype
Source: Front Endocrinol (Lausanne). 2021 Apr 23;12:628939. doi: 10.3389/fendo.2021.628939 (PMC8105402; doi:10.3389/fendo.2021.628939)
Supplement: Supplementary Table 1 — Clinicopathologic characteristics of patients with HER2-negative tumors. [file Table_1.docx]

| Table S1. Clinicopathologic characteristics of patients with HER2-negative tumors | | | | | |
| --- | --- | --- | --- | --- | --- |
| Variables | ER-/PR- | ER+/PR- | ER-/PR+ | ER+/PR+ |  |
| Age (years) | 61.93 ± 13.98 | 62.76 ± 14.24 | 62.09 ± 16.35 | 60.44 ± 15.53 | 0.561 |
| Follow-up time (median, months) | 26 (0-83) | 26 (0-83) | 35 (0-73) | 30 (0-83) | 0.753 |
| Race (n, %) |  |  |  |  | < 0.001 |
| Black | 191 (16.6) | 31 (14.7) | 23 (31.1) | 12 (9.8) |  |
| White | 882 (76.4) | 151 (71.6) | 45 (60.8) | 98 (80.3) |  |
| Other | 81 (7.0) | 29 (13.7) | 6 (8.1) | 12 (9.8) |  |
| Insurance (n, %) |  |  |  |  | 0.690 |
| No | 16 (1.4) | 4 (1.9) | 0 | 2 (1.6) |  |
| Yes | 1138 (98.6) | 207 (98.1) | 74 (100.0) | 120 (98.4) |  |
| Grade (n, %) |  |  |  |  | 0.007 |
| Undifferentiated | 35 (3.0) | 8 (3.8) | 1 (1.4) | 1 (0.8) |  |
| Poorly differentiated | 797 (69.1) | 136 (64.5) | 59 (79.7) | 74 (60.7) |  |
| Moderately differentiated | 133 (11.5) | 36 (17.1) | 8 (10.8) | 23 (18.9) |  |
| Well differentiated | 53 (4.6) | 8 (3.8) | 6 (8.1) | 5 (4.1) |  |
| Unknown | 136 (11.8) | 23 (10.9) | 0 | 19 15.6) |  |
| Tumor size (n, %) |  |  |  |  | 0.003 |
| T1 | 290 (25.1) | 52 (24.6) | 24 (32.4) | 41 (33.6) |  |
| T2 | 547 (47.4) | 103 (48.8) | 38 (51.4) | 58 (47.5) |  |
| T3 | 20 (17.6) | 22 (10.4) | 8 (10.8) | 15 (12.3) |  |
| T4 | 105 (9.1) | 29 (13.7) | 4 (5.4) | 5 (4.1) |  |
| Unknown | 9 (0.8) | 5 (2.4) | 0 | 3 (2.5) |  |
| Regional node status (n, %) |  |  |  |  | 0.002 |
| N0 | 911 (78.9) | 140 (66.4) | 61 (82.4) | 91 (74.6) |  |
| N1 | 171 (14.8) | 51 (24.2) | 8 (10.8) | 15 (12.3) |  |
| N2 | 39 (3.4) | 11 (5.2) | 1 (1.4) | 8 (6.8) |  |
| N3 | 25 (2.2) | 5 (2.4) | 2 (2.7) | 4 (3.3) |  |
| Unknown | 8 (0.7) | 4 (1.9) | 2 (2.7) | 4 (3.3) |  |
| TNM stage (n, %) |  |  |  |  | 0.023 |
| I-III | 1090 (94.5) | 194 (91.9) | 71 (95.9) | 115 (94.3) |  |
| IV | 56 (4.9) | 11 (5.2) | 3 (4.1) | 3 (2.5) |  |
| Unknown | 8 (0.7) | 6 (2.8) | 0 | 4 (3.3) |  |
| Chemotherapy (n, %) |  |  |  |  | 0.15 |
| No | 382 (33.1) | 74 (35.1) | 29 (39.2) | 52 (42.6) |  |
| Yes | 772 (66.9) | 137 (64.9) | 45 (60.8) | 70 (57.4) |  |
| Radiotherapy (n, %) |  |  |  |  | 0.632 |
| No | 660 (57.2) | 113 (53.5) | 38 (51.4) | 64 (50.8) |  |
| Yes | 494 (42.8) | 98 (46.4) | 36 (48.6) | 60 (49.2) |  |
| Type of surgery (n, %) |  |  |  |  | 0.200 |
| No | 63 (5.5) | 14 (6.6) | 2 (2.7) | 8 (6.6) |  |
| Lumpectomy | 477 (41.3) | 81 (38.4) | 40 (54.1) | 58 (47.5) |  |
| Mastectomy | 614 (53.2) | 116 (55.0) | 32 (43.2) | 56 (45.9) |  |
| Abbreviations: HER2 = human epidermal growth factor receptor 2; ER = estrogen receptor; PR = progesterone receptor. | | | | | |
