# Supplementary material for: Single Hormone Receptor-Positive Metaplastic Breast Cancer: Similar Outcome as Triple-Negative Subtype
Source: Front Endocrinol (Lausanne). 2021 Apr 23;12:628939. doi: 10.3389/fendo.2021.628939 (PMC8105402; doi:10.3389/fendo.2021.628939)
Supplement: Supplementary Table 2 — Clinicopathologic characteristics of patients with HER2-positive tumors. [file Table_2.docx]

| Table S2. Clinicopathologic characteristics of patients with HER2-positive tumors | | | | | |
| --- | --- | --- | --- | --- | --- |
| Variables | ER-/PR- | ER+/PR- | ER-/PR+ | ER+/PR+ |  |
| Age (years) | 56 (31-84) | 54 (39-77) | 49 (35-65) | 60 (40-77) | 0.188 |
| Follow-up time (median, months) | 28 (0-78) | 21 (4-73) | 25 (3-67) | 37 (4-64) | 0.682 |
| Race (n, %) |  |  |  |  | 0.808 |
| Black | 16 (24.6) | 4 (30.8) | 3 (30.0) | 2 (14.3) |  |
| White | 43 (66.2) | 7 (53.8) | 7 (70.0) | 10 (71.4) |  |
| Other | 6 (9.2) | 2 (15.4) | 0 | 2 (14.3) |  |
| Insurance (n, %) |  |  |  |  | 0.475 |
| No | 2 (3.1) | 0 | 2 (20.0) | 0 |  |
| Yes | 63 (96.9) | 13 (100.0) | 8 (80.0) | 14 (100.0) |  |
| Grade (n, %) |  |  |  |  | 0.075 |
| Undifferentiated | 1 (1.5) | 1 (7.7) | 0 | 0 |  |
| Poorly differentiated | 60 (92.3) | 11 (84.6) | 9 (90.0) | 9 (64.3) |  |
| Moderately differentiated | 1 (1.5) | 1 (7.7) | 1 (10.0) | 3 (21.4) |  |
| Well differentiated | - | - | - | - |  |
| Unknown | 3 (4.6） | 0 | 0 | 2 (14.3) |  |
| Tumor size (n, %) |  |  |  |  | 0.770 |
| T1 | 20 (30.8) | 2 (15.4) | 2 (20.0) | 3 (21.4) |  |
| T2 | 25 (38.5) | 6 (46.2) | 3 (30.0) | 5 (35.7) |  |
| T3 | 13 (20.0) | 3 (23.1) | 2 (20.0) | 4 (28.6) |  |
| T4 | 6 (9.2) | 2 (15.4) | 3 (30.0) | 1 (7.1) |  |
| Unknown | 1 (1.5) | 0 | 0 | 1 (7.1) |  |
| Regional node status (n, %) |  |  |  |  | 0.091 |
| N0 | 39 (60.0) | 9 (69.2) | 5 (50.0) | 7 (50.0) |  |
| N1 | 18 (27.7) | 3 (23.1) | 3 (30.0) | 1 (7.1) |  |
| N2 | 6 (9.2) | 0 | 2 (20.0) | 2(14.3) |  |
| N3 | 2 (3.1) | 1 (7.7) | 0 | 3 (21.4) |  |
| Unknown | 0 | 0 | 0 | 1 (7.1) |  |
| TNM stage (n, %) |  |  |  |  | 0.135 |
| I-III | 61 (93.8) | 10 (76.9) | 7 (70.0) | 13 (92.9) |  |
| IV | 3 (4.6) | 3 (23.1) | 3 (30.0) | 1 (7.1) |  |
| Unknown | 1 (1.5) | 0 | 0 | 0 |  |
| Chemotherapy (n, %) |  |  |  |  | 0.620 |
| No | 10 (15.4) | 2 (15.4) | 0 | 2 (14.3) |  |
| Yes | 55 (84.6) | 11 (84.6) | 10 (100.0) | 12 (85.7) |  |
| Radiotherapy (n, %) |  |  |  |  | 0.344 |
| No | 33 (50.8) | 9 (69.2) | 6 (60.0) | 5 (35.7) |  |
| Yes | 32 (49.2) | 4 (30.8) | 4 (40.0) | 9 (64.3) |  |
| Type of surgery (n, %) |  |  |  |  | 0.195 |
| No | 3 (4.6) | 3 (23.1) | 2 (20.0) | 1 (7.1) |  |
| Lumpectomy | 25 (38.5) | 3 (23.1) | 1 (10.0) | 5 (35.7) |  |
| Mastectomy | 37 (56.9) | 7 (53.8) | 7 (70.0) | 8 (57.1) |  |
| Abbreviations: HER2 = human epidermal growth factor receptor 2; ER = estrogen receptor; PR = progesterone receptor. | | | | | |
