# Supplementary figures and images for: Single Hormone Receptor-Positive Metaplastic Breast Cancer: Similar Outcome as Triple-Negative Subtype
Source: Front Endocrinol (Lausanne). 2021 Apr 23;12:628939. doi: 10.3389/fendo.2021.628939 (PMC8105402; doi:10.3389/fendo.2021.628939)

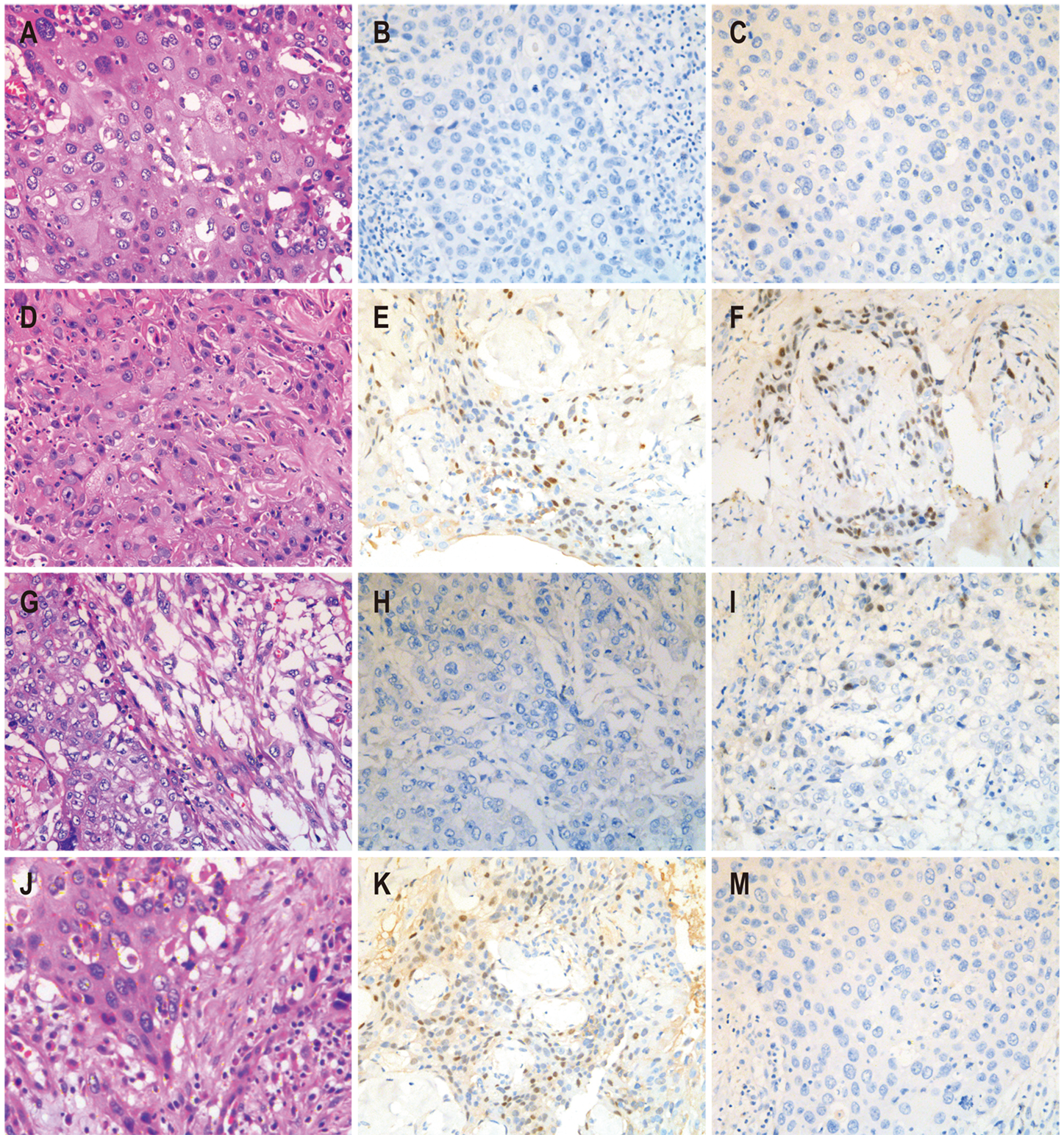

Supplement: Supplementary Figure 1 — Four MBC patients with different estrogen receptor (ER)/progesterone receptor (PR) phenotype. a, H&E, ×200; b, ER (-),×200; c, PR (-),×200; d, H&E, ×200; e, ER (+),×200; f, PR (+),×200; g, H&E, ×200; h, ER (-),×200; i, PR (+),×200; j, H&E, ×200; k, ER (+),×200; m, PR (-),×200. [file Image_1.tif]
